# Our Position on the Vivisection Question

**Published:** 1898-02-12

**Authors:** 


### Our Position on the Vivisection Question.

An article in our issue for January 20th has raised the indignation of the *Star*, and has also brought upon us a long letter from the secretary of the London Anti-Vivisection Society.

We do not in any way feel called upon to print this letter, but we have no objection to state our views in regard to vivisection, and if these views, which we fancy are pretty widely held by men of experience and education, do not commend themselves to Mr. Trist and the editor of the *Star*, we must, more or less sorrowfully, put up with our misfortune.

We take it that there are many questions which are best decided in accordance with that form of compromise which goes by the name of common sense, and that of these vivisection is a marked example. Abstract principles do not touch the question at issue, and cannot touch it so long as the world remains entirely at loggerheads as to what forms of cruelty are right and what are wrong. None can be more opposed than we are to wanton cruelty, none can be more anxious than we are to alleviate suffering and assuage pain, yet we recoil from the crude assumption that it is wrong to inflict pain with the object of increasing our knowledge of disease—a doctrine which would condemn the human race, and even animals themselves, to much suffering, from which they could be relieved by aid of the knowledge so obtained; and we think that, safeguarded as the practice now is by law, the country is enabled to get much of the good which can be derived from vivisection, with but little—practically none—of the evils which might attend its unrestricted use.

There used to be a form of vivisection from which all men of humanity revolted; vivisection done wantonly, done without anæsthetics for mere purposes of demonstration, or for practice in operative work. But all this is absolutely a thing of the past. At the present time vivisection is entirely illegal in this country except by a few licensed persons in a few licensed premises under Government inspection, and even then under very definite restrictions. That these restrictions are effectual the public may feel well assured by the fact that we do not hear of prosecutions for their infringement. We have the greatest admiration for the ingenuity with which the officials—we hope we shall not be thought discourteous if we say the paid officials—of the societies which interest themselves in this matter do their work. The catchy little pamphlets, the newspaper paragraphs, the endless letters with which the Press is inundated, all betoken unbounded energy and push. We need hardly say, however, that, as a means of attracting attention to the subject, a single case in the police-courts, a single proof that the law is being infringed, would be worth tons of such rubbish—but that case is not forthcoming. This, however, is nothing to the thorough-going anti-vivisector. To him any experiment upon an animal is wrong; it is an unholy thing; it is a thing to be stopped at all cost, and he asks that it shall be by law abolished absolutely and for ever.

The life of a medical man, however, makes him look upon this question from a more serious standpoint. He sees around him a world of suffering. He spends his time endeavouring to cure disease and lighten pain, and if it be said by the thoughtless that he is paid to do it that in no way alters the fact that his main aim in life, whether for conscience or for cash, is the alleviation of the suffering which he finds around him. When, however, he looks out upon the world he finds something else; he finds on every hand a perfect ocean of cruelty, and he not unnaturally asks why he should refuse to do his duty, should hold back from curing his patient, should hesitate to find out the nature of the diseases which he is contending with, at the behest of a small group of people who choose to set up a dogma in regard to cruelty which is obviously opposed to the standard accepted as a general guide to conduct in the world in which he lives; a world of sport, of coursing, and of pigeon shooting, a world in which tender women bait mousetraps, so that the poor mice may be kept impaled for hours before they die, in which people go out shooting and ferreting for amusement, in which dogs are trained to worry rats, and rats are caught and kept and sold for the purpose of being worried, and a world in which the cruelties of the shambles go on unchecked.

We would gladly see both cruelty and pain vanish from the world. But there is a pain of disease as well as a pain of vivisection, and the latter is a mere infinitesimal trifle compared with the widespread suffering it is meant to combat. If people are sincere in their war against cruelty, let them begin by abolishing what is wanton; but not until disease, and all the torture which it causes, is infinitely less prevalent than it is at present, would we prohibit the performance of experiments on animals done with the object of relieving the sufferings of men.

